# Bacterial vaginosis in microscopic examination of Pap smears in patients with high-risk HPV is associated with viral persistence and cytological progression in a longitudinal study

**DOI:** 10.1186/s13027-026-00734-x

**Published:** 2026-01-29

**Authors:** Lara Linz, Nina Zachau, Sibylle Spieth, Patrick Finzer

**Affiliations:** 1 Institut fuer Medizinische Mikrobiologie und Krankenhaushygiene, Universitaetsklinikum Duesseldorf, Heinrich-Heine Universitaet Duesseldorf, Duesseldorf, Germany; 2 CBT Bonn, Zytologie und Pathologie, Bonn, Germany

**Keywords:** Bacterial vaginosis, Pap smears, High-risk HPV, HPV-persistence, Cytological progression

## Abstract

**Background:**

The vaginal microbiome is increasingly studied using sequencing approaches; however, the exact bacterial community structure in vaginal diseases, e.g. bacterial vaginosis (BV), remains unclear. Vaginal Pap smears routinely obtained in preventive screening programs can also be evaluated with high accuracy regarding microbial colonization by means of microscopy.

**Methods:**

This longitudinal retrospective case control study aims to investigate the relationship between microscopic vaginal flora observed in Pap-smears and Human Papilloma Virus (HPV) clearance, persistence or progression using HPV-PCR tests and the results of the Pap-smears. Two cohorts of high-risk HPV positive patients were formed. Cohort A (*n* = 136) was observed over a two-year time period 2020–2022, while cohort B (*n* = 160) was observed over a one-year period from 2021 to 2022. Each case group consisted of BV-positive patients that were compared to BV negative patients as controls.

**Results:**

It was found that the case groups in both cohorts had a higher HPV persistence rate and a higher rate of progression at follow-up. Lactobacillus flora was consistently associated with favorable outcomes, particularly higher HPV clearance rates across both cohorts and the entire observation period. In contrast patients with bacterial vaginosis (BV) were associated with significantly increased risk of HPV progression, especially in Cohort B, supporting its role as risk factor in the development of precancerous cervical lesions.

**Conclusion:**

The integration of microscopic flora assessment into screening programs for cervical cancer may help to improve risk stratification in HPV positive women. Our findings suggest that, BV-positive patients may benefit from a closer follow-up period and earlier therapeutic intervention.

**Clinial trial number:**

Not applicable.

**Supplementary Information:**

The online version contains supplementary material available at 10.1186/s13027-026-00734-x.

## Background

The Human Papilloma Virus (HPV) has a high global prevalence [1]. In 2018 approximately 690,000 cases of cancer related to HPV were diagnosed [2]. Cervical cancer is the most common HPV related cancer (about 80% of all HPV related cases) [2].

Although over 90% of HPV-infections are temporary and resolve within one to two years [3], HPV-DNA is found in nearly all cervical cancer lesions [4]. The persistence of HPV may result in cervical intraepithelial neoplasia (CIN), that can further develop into cervical cancer [5]. While an HPV infection is a necessary precondition for the development of cervical cancer [4], additional factors appear to contribute to the development of cervical cancer.

The vaginal microbial community might be one of these factors providing protective as well as pathogenic impact for the development of cervical cancer. Various classification systems are used to describe the composition of the vaginal flora depending on the utilized diagnostic method. E.g. for next generation sequencing (NGS) data the vaginal community state types (CST) are used to describe vaginal microbiota [6, 7] and the Nugent score is applied when looking at Gram-stained smears [8]. A healthy vaginal microbiome is, in most cases dominated by Lactobacillus species, which help to maintain a low vaginal pH and thereby protect the mucosa [9]. A reduction of Lactobacilli can result in a more diverse microbiome called a mixed flora which favors pathological development [10].

Bacterial Vaginosis (BV) in general describes a state of dysbiosis with a loss of *Lactobacillus* dominance and a predominance of anaerobic bacteria such as *Gardnerella vaginalis* and is seen as pathological [11, 12] not only because it leads to clinical symptoms like excess grayish vaginal discharge and unpleasant vaginal odor but also because of its potential role in increasing susceptibility to HPV infection and persistence [13]. BV is commonly diagnosed using the Amsel criteria, requiring three of the following: homogenous greyish vaginal discharge, elevated vaginal pH (> 4.5), a positive whiff test (amine odor with potassium hydroxide) and presence of clue cells on microscopic examination [14, 15].

The relationship between composition of the vaginal microbiome and HPV-related outcomes has been studied in recent years. It has been demonstrated that Lactobacillus dominated flora might be a protective factor against the development of cervical cancer [16]. Conversely, BV has been associated with an increased risk for the development of CIN [17]. In a longitudinal study, microbiome sequencing and the development of a molecular BV tool demonstrated that BV might significantly affect HPV persistence [18].

However, the commonly used 16 S amplicon-based sequencing has notable limitations, a putative bias by PCR amplification and the associated restrictions in the assignment of the bacteria found to the respective taxonomic level, which also affects estimates of their relative abundances [19]. In addition, the NGS technique used is cost-intensive and not widely used in routine diagnostics [20].

The composition of the vaginal microbiota can be assessed by microscopical examination, which can also be done during the microscopic assessment of pap smears [21]. This microbiological approach is widely accessible and enables a semi-quantitative evaluation of the vaginal flora based on morphology, distribution and density of bacterial forms [10]. Predominance of small coccobacilli for example can be seen in patients with BV [22]. Another diagnostic criterion are clue cells, which are squamous epithelial cells with blurred borders caused by dense adherence of small coccobacilli; these cells are pathognomonic for BV and a central key finding [15]. These aspects can also be classified according to the Lactobacillary Grades proposed by Donders [21]. Therefore, Pap smears allow the qualitative interpretation of the flora with clinical and diagnostic significance, which appears to correlate well with NGS data [23]. Particularly in retrospective studies, they serve as valuable source for correlating vaginal flora with CIN and help to assess host microbe interaction in the cervicovaginal environment [23]. However, to our knowledge, there are no longitudinal studies examining cytological and microbiological examination of pap smears with HPV.

This study aims to investigate the prevalence of BV, its potential impact on high-risk (HR) HPV persistence and dysplastic progression using Pap-smear records. The objective is to gain a deeper understanding of the relationship between vaginal microbiome and HPV based on methods other than NGS.

## Methods

### Study cohort

The study group was retrospectively generated from patients of Cyto Labor Bonn who had pap smears between the years 2020 and 2022. Patients for the case group were included according to the following criteria: (1) Bacterial vaginosis at the beginning of the observation period (BV+) (2), positive high-risk (HR) HPV-PCR test result at the beginning of the observation period and (3) one Pap smear for each year of the observation period as well as (4) HPV-PCR follow-up data. Two case cohorts were formed: The first one from 2020 to 2022 (Cohort A); the second one from 2021 to 2022 (Cohort B).

The control group was constituted by patients who did not show BV at the beginning of the observation period (BV-) but were positive in the HR HPV-PCR test. These patients also required annual Pap smear results. The first 15 patients from each month were scanned for patients fitting the criteria of the control group until the same number of patients as in the case group was acquired.

This retrospective study was approved by the Ethics Committee of the Faculty of Medicine at Heinrich Heine University Duesseldorf (Study: 2024 − 2988 – “CytoHPV”) and all methods were carried out in accordance with the relevant guidelines and regulations.

### Pap smears

Cervical cytology samples were collected by trained gynecologists using two different methods: conventional Pap smear and the liquid-based BD Sure Path technique, depending on the setting and available resources. The conventional specimens were immediately smeared onto a glass slide and fixed. In the laboratory they were stained using the Papanicolaou method on the Sakura Prisma staining system. The Sure path samples were preserved in the BD Sure path collection vial and then processed using the BD PrepMate system. First the solution was homogenized and filtered to remove residue. Subsequently the remaining cells are transferred onto a glass slide and finally stained using the Papanicolaou staining protocol on the Sakura Prisma staining system:

For Papanicolaou staining the slides were fixed in 99.8% ethanol, followed by rinsing in distilled water. Nuclear staining was performed using Hematoxylin in two steps. To rinse off any excess stain the slides were rinsed with tap water and ethanol-water mixtures to prepare the samples for cytoplasmic counterstaining. To highlight keratinized cells Orange G was applied, followed by dehydration steps and polychromatic staining with Eosin Azure to differentiate non-keratinized cells. The Final dehydration was carried out with absolute ethanol, followed by clearing xylene-ethanol and pure xylene to preserve the stained preparation an enhance transparency for microscopic evaluation. By using an automated protocol, consistent and reproducible staining quality was ensured.

### Microscopy

Each of the Pap smears was examined by a trained cytological pathologist of the Cyto Labor Bonn. The Pap results were classified according to the Muenchner Nomenklatur III for the gynecological cytodiagnostics of the cervix (MN) [24] (see Supplementary Table 1).

The classification of vaginal microbiota was based on cytomorphological criteria. Four distinct types were identified: (1) A Lactobacillus dominated flora characterized by monoformus long rod-shaped bacteria that are evenly distributed and predominate the background (2). A mixed flora characterized by a reduced number of Lactobacilli and a greater number of other bacteria like short rods, cocci or coccobacilli (3). A coccal flora dominated by cocci with almost no detectable Lactobacilli (4). The detection of clue cells, which are superficial squamous epithelial cells with blurred borders due to dense bacterial coating, which is why we have named this group BV positive (BV+).

### HPV-PCR

Cervical specimens were also tested for HR HPV-DNA using BD Onclarity™ HPV Assay on the BD COR™ System. This is a qualitative real-time PCR test that provides individual genotyping results for HPV 16,18 and 45, and pooled results for the remaining HR types (e.g., 31, 33/58, 52, 35/39/68, 51, and 56/59/66). Virus -testing was performed at Zotz-Klimas MVZ-Centrum, Duesseldorf. For this study, samples were processed according to manufacturer’s instructions. Results were interpreted using predefined cycle threshold (Ct) values.

### Data analysis

The data were analyzed with SPSS version 29.0.1.0. To summarize the baseline characteristics of the study participants descriptive statistics were used. To analyze the difference in outcomes (clearance, persistence, progression) between the different groups the odds ratios and Mann-Whitney U tests were calculated. A p-value of < 0.05 was considered statistically significant.

## Results

### Study cohorts

**Fig. 1:**
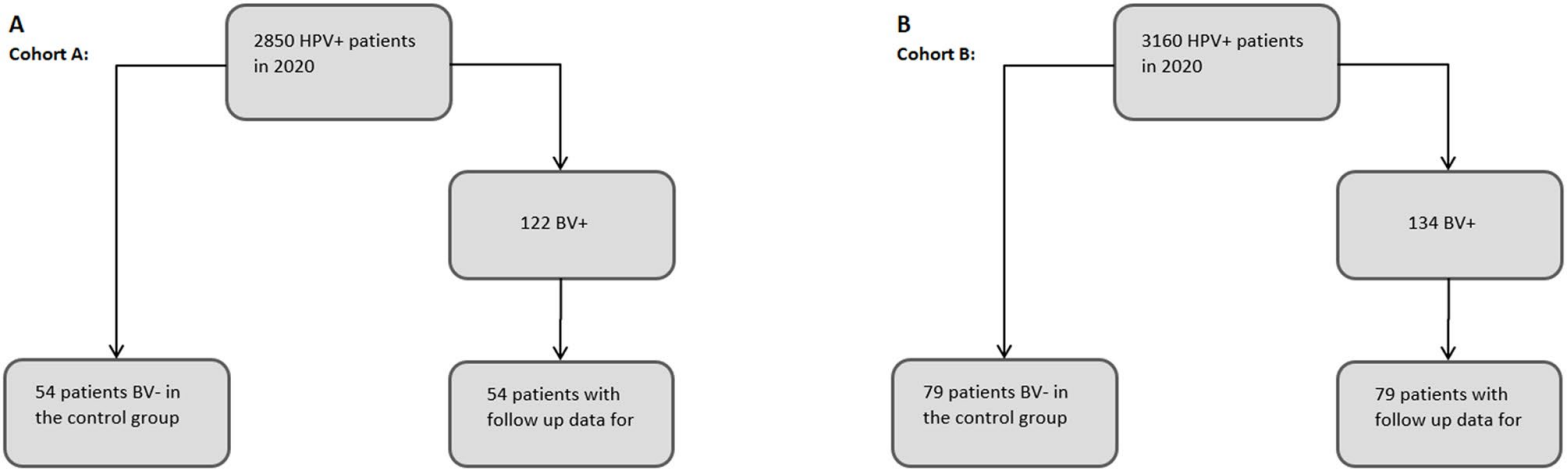
Flow chart for the creation of Cohorts A and B. **A**: Cohort A: Flowchart of patient selection. From 2,850 h HPV-positive patients in 2020, 122 patients were identified as BV+. Of these, 54 patients had follow-up results for 2021 and 2022. A control group of 54 BV- patients was also included. **B**: Cohort B: Flowchart illustrating the selection of patients. Of 3,160 h HPV-positive patients, 134 were identified as BV+. Among these, 79 patients had follow-up data for 2022. A control group of 79 BV- patients was also included for comparison

**Fig. 2:**
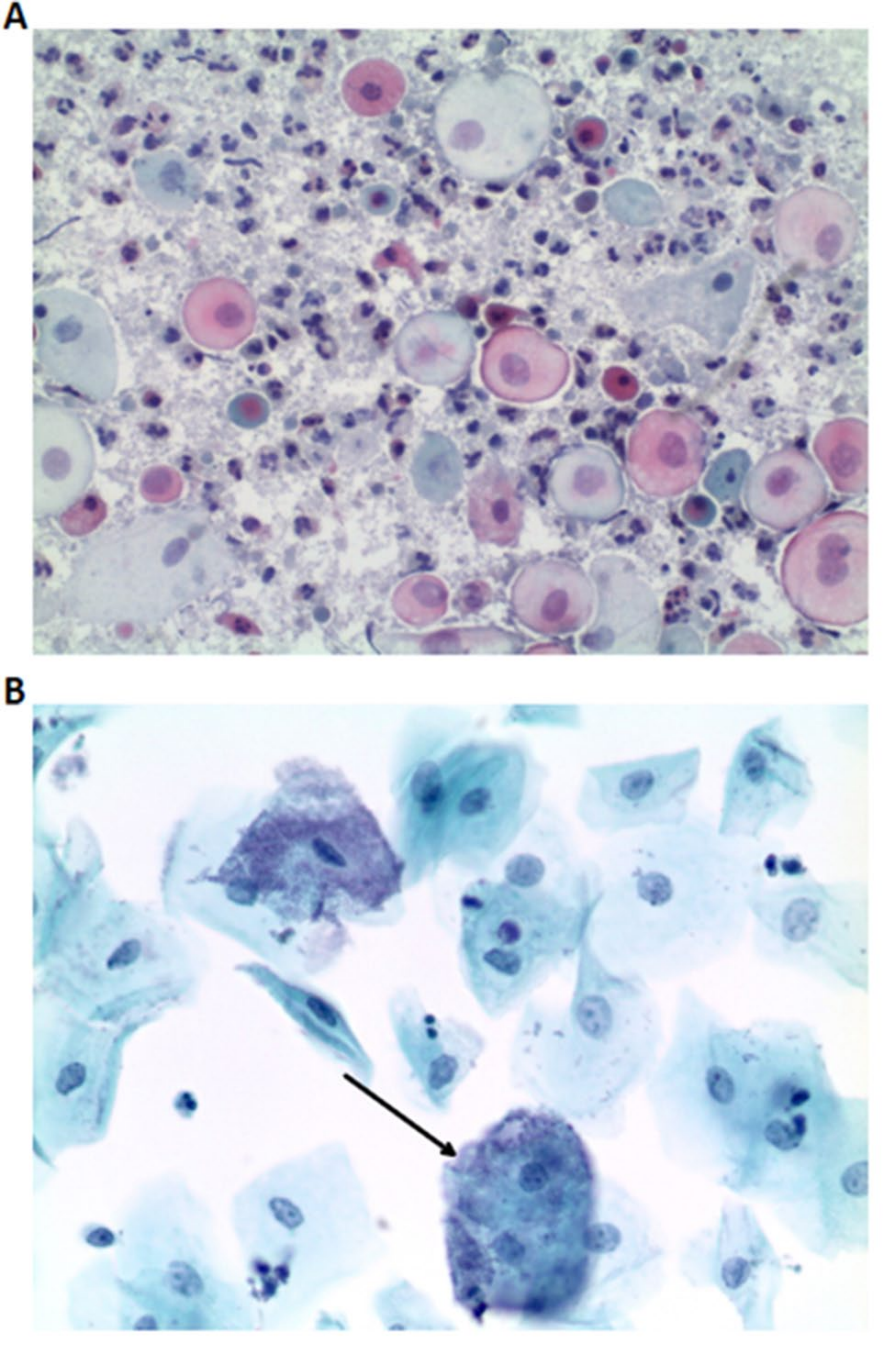
Cytological examination of Pap-Smears. **A**:10x magnification of a Coccus-dominated flora. **B**: 40x magnification of a Surepath smear, BV+

To investigate the relationship between HR HPV infection and the vagino-cervical microbiome over the course of the disease, a study cohort was formed that was positive for bacterial vaginosis (BV+) and HR HPV in 2020 and in which cytological and virological findings were also available for the two subsequent years (Cohort A). From 2,850 h HPV-positive patients in 2020, 122 patients were identified as BV+ (4,2%). Of these, 54 patients had follow-up results for 2021 and 2022. A control group of 54 BV- patients was also included (see Fig. 1A). Examples for Pap-smears are shown in Fig. 2.

To control the results, a second cohort was generated which was BV + and HR HPV-positive in 2021 and had cytological and virological findings in the following year (Cohort B). Of 3,160 h HPV-positive patients, 134 were identified as BV+ (4,2%). Among these, 79 patients had follow-up data for 2022. A control group of 79 BV- patients was also included for comparison. (see Fig. 1B).

In Cohort A, the mean age in the BV + group was 45.19 years, while in the control group it was 48.57 years. The Levene’s Test demonstrated that the variance in both groups was the same (*F* = 2.528, *p* = 0.115). Therefore, the results were interpreted on the assumption of equal variances. The independent t-test did not reveal a statistically significant difference between the BV + group (*M* = 45.19, *SD* = 9.543) and the BV − group (*M* = 48.57, *SD* = 10.962), *t*(106) = − 1.713, *p* = 0.090. The mean age difference was − 3.389 years [− 7.310–0.532] (see Table 1).

**Table 1:** Sociodemographic data

Cohort A	Mean (years)	Standard deviation	Mean age difference	Levene-Test	T-Test
BV+	45.19	9.543			
BV-	48.57	10.962	-3.389 [-7.310–0.532]	F = 2.528 *p* = 0.115	t(106)=-1.713 *p* = 0.090

Cohort B	Mean (years)	Standard deviation	Mean age difference	Levene-Test	T-Test
BV+	45.42	9.867	1.582 [-1.514–4.678]	F = 0.324 *p* = 0.570	t(156) = 1.009 *p* = 0.314
BV-	47.00	9.836			

The mean age of Cohort B in the case group was 45.42 years and 47.00 years in the BV- group. Levene’s test indicated that the variances between the two groups are similar (F = 0.324, *p* = 0.570). Therefore, the t-test results assuming equal variances were interpreted. The independent samples t-test revealed no significant difference in age between the BV + group (M = 45.42, SD = 9.867) and the BV- group (M = 47.00, SD = 9.836), t(156) = 1.009, *p* = 0.314. The mean difference in age was 1.582 years [-1.514–4.678] (see Table 1). Both cohorts showed approximately the same age distribution and could therefore be used well as respective controls.

### Higher rate of HR HPV detection in case groups at follow-up

**Fig. 3:**
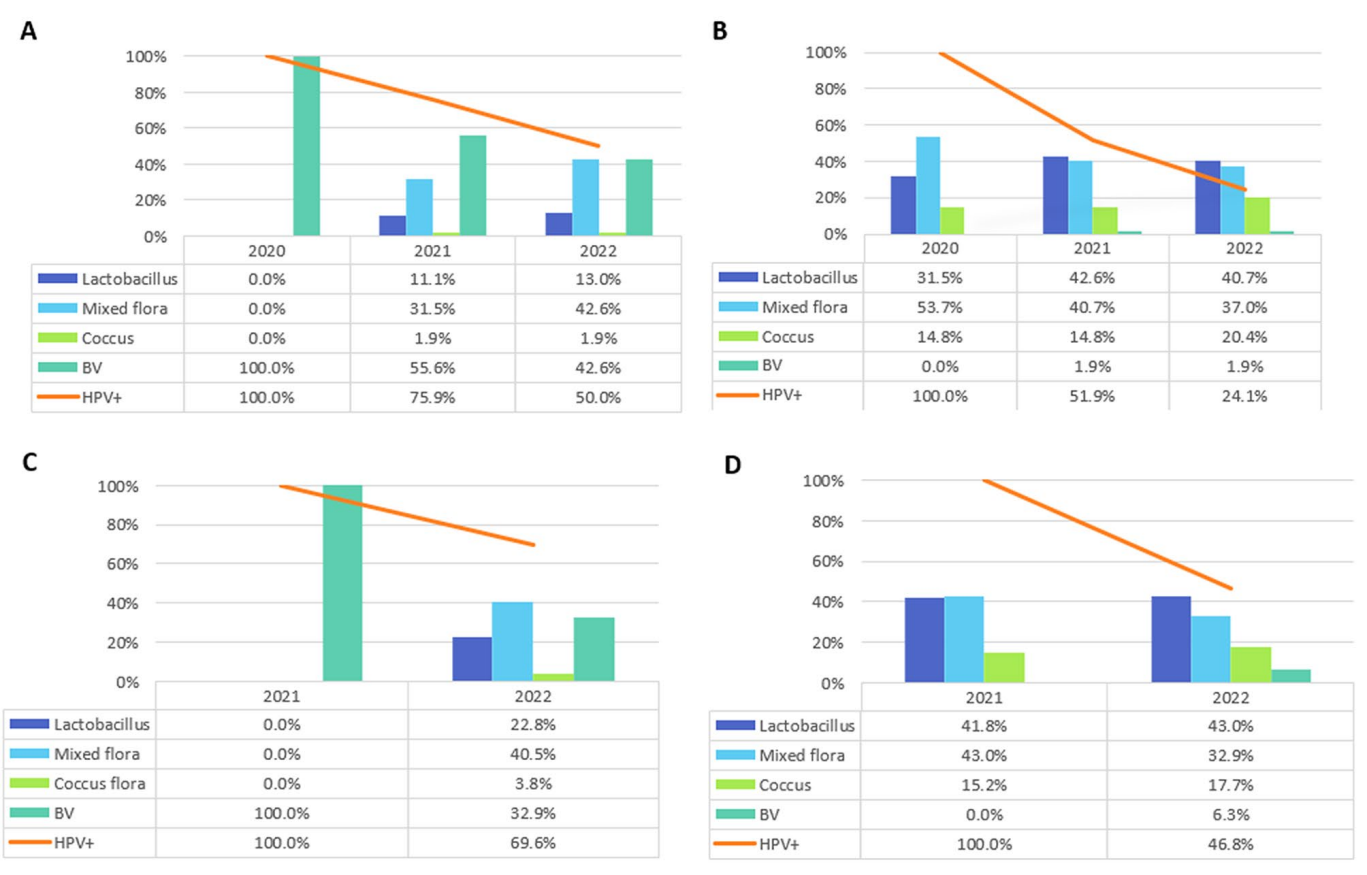
Cohort A: The course of flora types and HPV-detection. **A**: Cohort A BV + patients regarding flora types and HPV status from 2020–2022. **B**: Cohort A BV- patients regarding flora types and HPV status from 2020–2022. **C**: Cohort B BV + and BV- patients regarding flora types and HPV status from 2021. **D**: Cohort B BV + and BV- patients regarding flora types and HPV status from 2022

In the BV + group in Cohort A, BV remained the dominant flora type throughout the observation period. In 2021 it accounted for 55.6% of samples and 42.6% in 2022. Lactobacillus remained low at 11.1% from 2021 to 13.0% in 2022. Mixed flora showed a sharp increase to 31.5% in 2021 and 42.6% in 2022. Coccus flora was detected at low levels (1.9%) in the later years (see Fig. 3A).

In the BV − group of Cohort A, Lactobacillus was the predominant flora type, found in 31.5% of samples in 2020, peaking at 42.6% in 2021, and slightly declining to 40.7% in 2022. Mixed flora remained consistently high (53.7% in 2020, 40.7% in 2021, and 37.0% in 2022), while Coccus flora was present in moderate amounts (14.8–20.4%). BV was rare in this group, with a maximum prevalence of only 1.9% in 2021–2022 (see Fig. 3B).

In 2021 75.9% of patients in the case group remained HR HPV+, compared to 51.9% in the control group. In the final year of the observation period (2022) 50.0% of the case group remained HR HPV + whereas a total of 24.1% of patients in the control group were HR HPV+. The most prevalent HPV type in 2020 was HPV 16, followed by 31 and 18 (see Supplementary Table 2).

In the BV + group of Cohort B the microbial composition changed notably between 2021 and 2022. In 2022 BV declined to 32.9%, while Mixed flora emerged as the dominant type with 40.5%. Lactobacillus was seen in 22.8% of patients and Coccus flora in 3.8% (see Fig. 3C).

In the BV- group of Cohort B the flora remained more stable. Lactobacillus was the most prevalent species in both years with a slight increase from 41.8% to 43% in 2022. Mixed flora showed a modest decline from 43% to 32.9% during the same period. Coccus flora was detected at 15.2% in 2021 and 17.7% in 2022. A few patients also developed BV in 2022 (6.3%) (see Fig. 3D). In 2022 69.6% of patients in the case group of Cohort B had a persistent HR HPV infection, compared to 46.8% in the control group. The most prevalent HPV type in 2021 was HPV 16, followed by HPV 31 (see Supplementary Table 3).

In both cohorts, the picture is thus the same: the BV + flora is more variable than the BV-, and HPV detection is higher in the BV + group than in the BV- at follow-up.

### Higher rate of cytological progression in case groups at follow-up

**Fig. 4:**
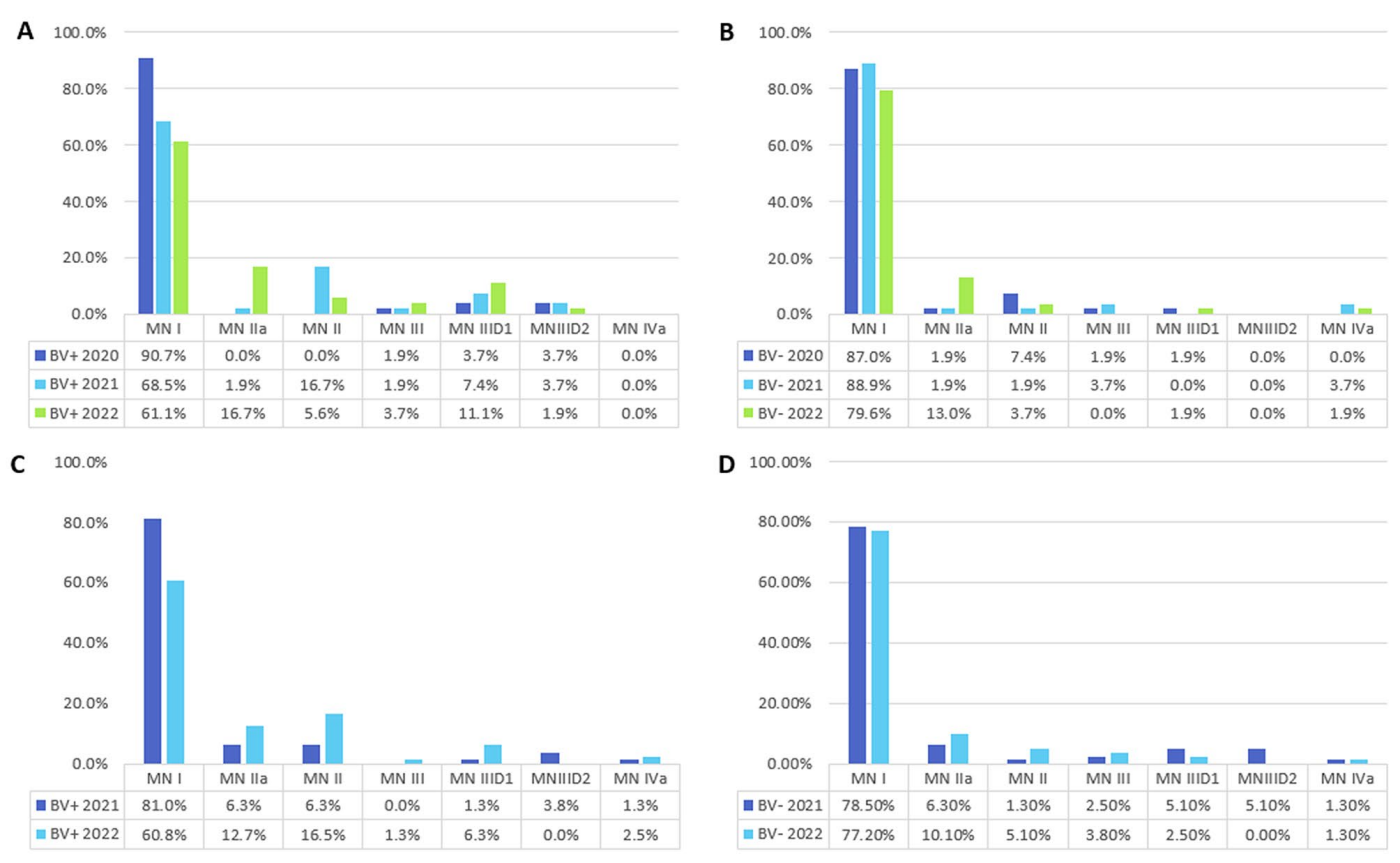
Cytological findings over time. **A**: Cohort A Comparison of the result of the Pap smear according to MN III from 2020–2022. **B**: Cohort A BV- Comparison of the result of the Pap smear according to MN III from 2021–2022. **C**: Cohort B BV + Comparison of the result of the Pap smear according to MN from 2021–2022. **D**: Cohort B BV- Comparison of the result of the Pap smear according to MN from 2021–2022

Cytological assessment changed over time. Most cases in Cohort A, both in BV + and BV-, were classified as MN I and their portion decreased at follow-up. It was noticeable that there were more MN II a and MN III D1 cases in the BV + group than in the BV- group at follow-up examination (Fig. 4A + B). The same observation could also be made for cohort B, with an additional increase in MN II in the BV + group (Fig. 4C + D).

As primary outcome of this study we assessed the change in Pap smear assessment according to MN combined with the result of the HPV-PCR Test over the entire observation period for each cohort, categorized as: (1) clearance: HPV negative after the entire observation period (2), persistence: HPV positive with a consistent result in the Pap smear or (3) progression: HPV positive with a worsening Pap result after the entire observation period. Worsening is defined as a progression to a higher category according to the MN [24].

**Fig. 5:**
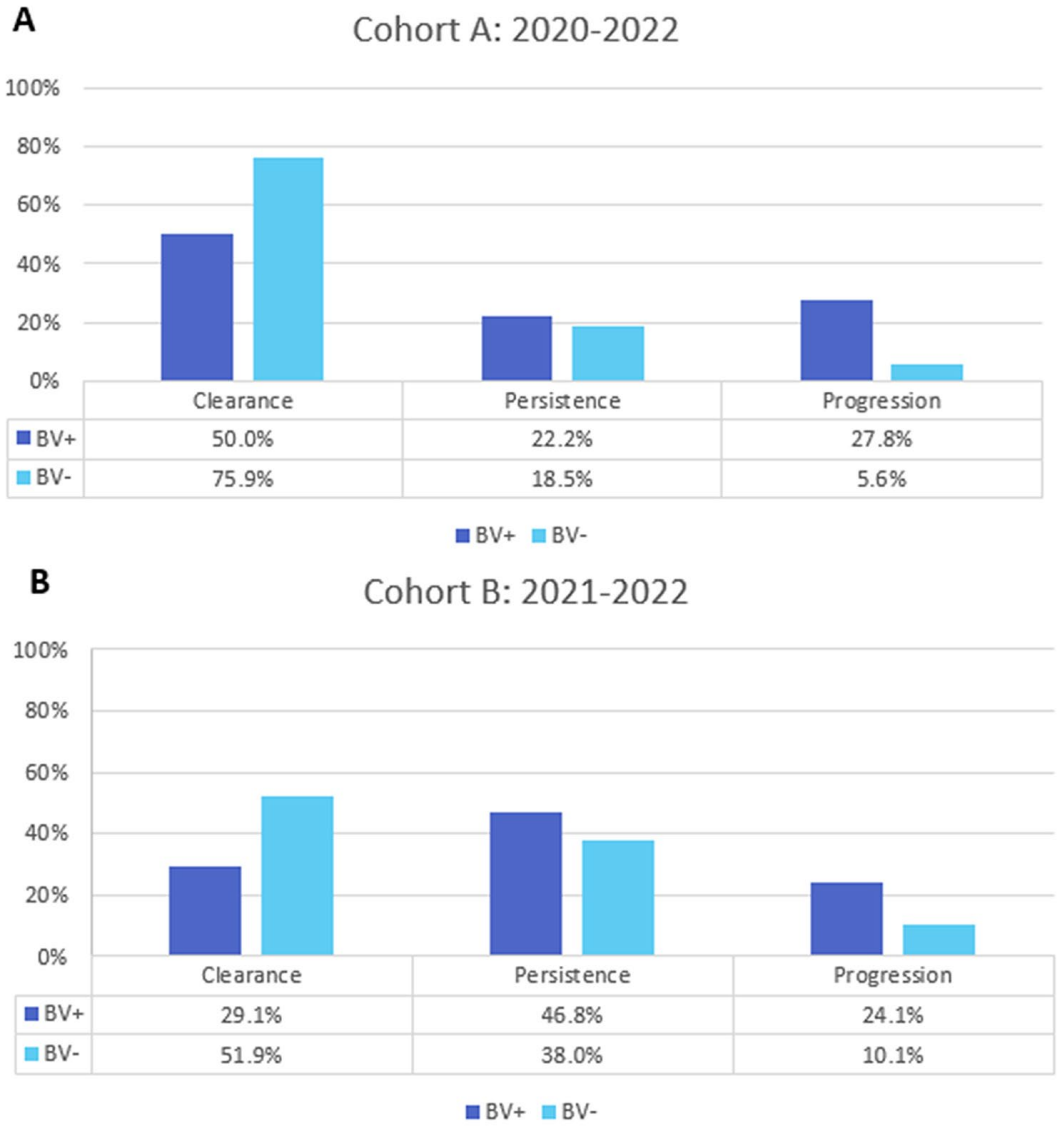
Primary outcome: Cohort A and Cohort B. **A**: Cohort A primary outcome analysis 2020–2022. **B**: Cohort B primary outcome analysis 2021–2022

Over the entire observation period of Cohort A the progression in the case group was 22.2% points higher than in the control group. Furthermore, the persistence rate was 3.7% points higher than in the control group. As anticipated, the clearance rate was lower than in the control group, with a difference of 25.9% points (see Fig. 5A).

In Cohort B 29.1% of patients in the BV + group showed clearance compared to 51.9% in the BV- group in 2022. This represents a 22.8% points higher clearance rate in the BV- group. Persistence was slightly more prevalent in the BV + group with 46.8% compared to 38.0% in BV- group. In the case group 24.1% of patients progressed compared to 10.1% in the control group. In cohort B, this translates into a 14% points higher progression rate in the BV + group (see Fig. 5B).

**Fig. 6:**
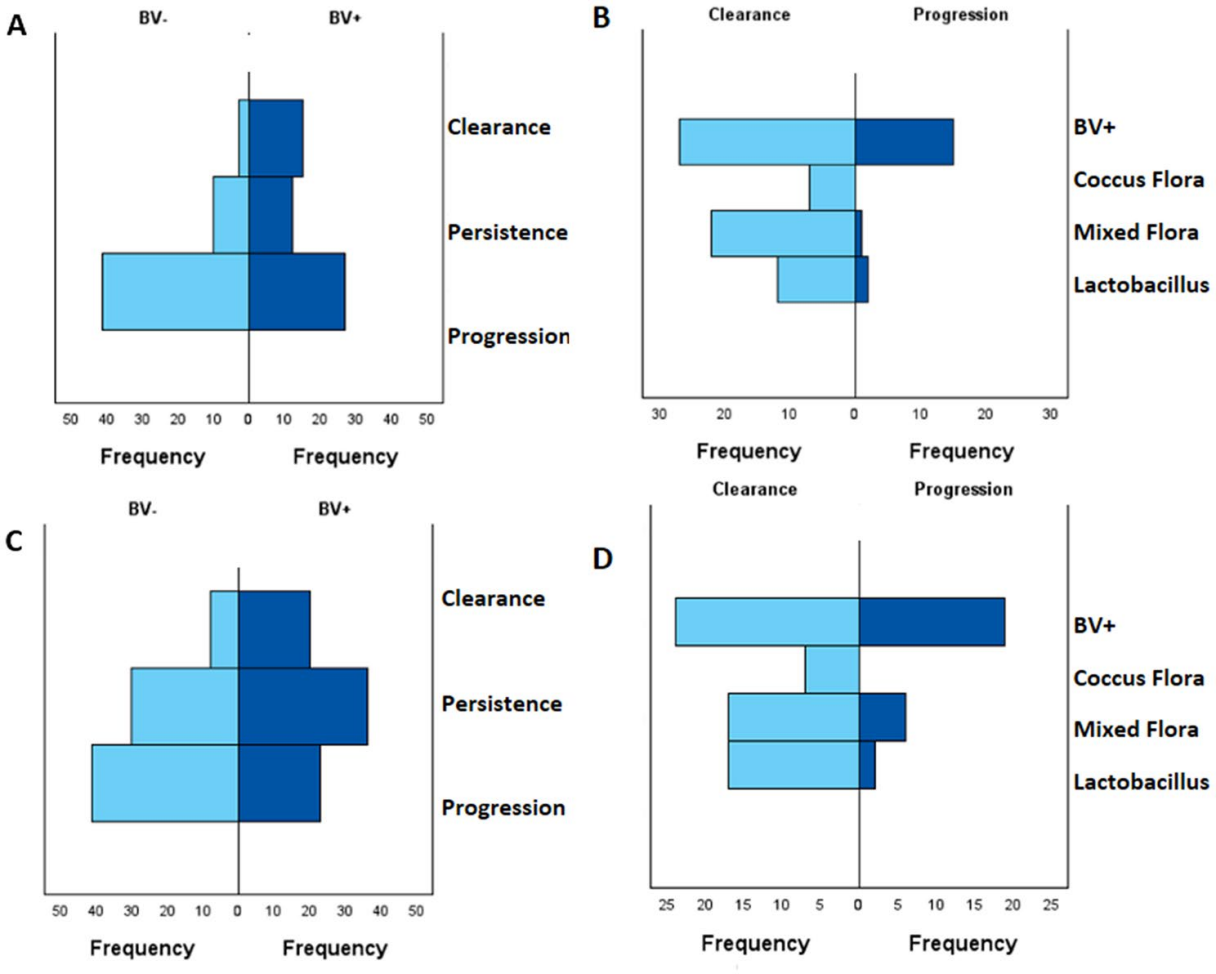
Comparison of primary outcomes and HPV-Detection and flora type. **A**: Cohort A: Mann-Whitney U test comparing primary outcomes from 2020–2022 between BV- (mean rank = 46.44) and BV+ (mean rank = 62.56); mean rank in the BV + group (*p* = 0.002). **B**: Cohort B: primary outcomes from 2021–2022 between BV- (mean rank = 68.53) and BV+ (mean rank = 90.47) **C**: Cohort A: Mann-Whitney U test comparing the flora types of the clearance group (mean rank = 39.96) versus the progression group (mean rank = 56.86) with a statistically significant result from 2020-2j022 (*p* = 0.006). **D**: Cohort B: Mann-Whitney U test comparing the flora types from 2021–2022 of the clearance group (mean rank = 41.89) versus the progression group (mean rank = 57.59) with a statistically significant difference (*p* = 0.006)

The Mann-Whitney U test for cohort A indicated a statistically significant difference between patients of the case and control group over the entire observation timeline. The BV + group had a mean rank of 62.56 compared to 46.44 in the control group with U = 1893.0 and *p* = 0.002 (see Fig. 6A). These findings indicate that patients in the case group who had BV at the time of HR HPV diagnosis were more likely to show progression in Pap smear results. The odds ratio for progression with a BV + flora is 7.593 [2.005–28.747]. Suggesting a higher rate of progression for the case group.

In Cohort B, the Mann-Whitney U test was conducted to determine the clearance, persistence, and progression rate of the two groups. The results of cohort B indicated a statistically significant difference in outcome between the case group and the control group (*p* = 0.001 U = 3987.500) with a mean rank in the BV + group of 90.47 and in the control group of 68.53 (see Fig. 6C). This suggests that the patients in the BV + group were more likely to experience an unfavorable outcome. The odds ratio for progression in the year 2021 for patients with a BV + flora is statistically significant with an odds ratio of 4.057 [1.542–10.667]. This suggests that being BV + is a risk factor for progression.

### Lactobacilli are associated with HPV clearance

In a complementary analysis, patients were reclassified into a clearance and progression group based on their HPV outcome over the entire follow-up period. A MWU test comparing the flora types of the clearance group (mean rank 39.96) versus progression group (mean rank 56.86) revealed a statistically significant difference over the observation period in Cohort A from 2020 to 2022 (*p* = 0.006; U = 852.5), with the progression group exhibiting a less favorable vaginal flora (see Fig. 6B).

To assess if the progression group of Cohort B was more prone to a pathological flora type, we performed another Mann-Whitney U test. The results indicated the progression group is associated with a more pathological flora type (*p* = 0.006; U = 1177.000). The mean rank in the clearance group was 41.89 compared to 57.59 for the progression group of Cohort B (see Fig. 6D).

## Discussion

The longitudinal design of our study, comprising a two-year follow-up period for cohort A and a one-year follow-up period for cohort B, enabled us to investigate the role of vaginal flora - particularly BV – in the clearance or progression of HR-HPV infected patients.

A comparable longitudinal study has already shown a link between bacterial vaginosis (BV) and HR-HPV clearance or progression [25]. However, important methodological and demographic differences must be considered when comparing these results. Usyk et al. used data from a vaccine trial, resulting in a substantially younger study population with a mean age of 23 years [18, 25], whereas the mean age of participants in our cohort was 48 years. This age difference has significant gynecological implications, as a considerable proportion of women in our cohort were peri- or postmenopausal, a physiological state known to be associated with marked changes in the vaginal microbiome [26]. However, the age distribution of our study population more accurately reflects the target group of routine cervical cancer screening and prevention programs. Furthermore, Usyk et al. focused exclusively on progression to CIN 2 or higher [25]. In contrast our study evaluated distinct cytological outcomes, allowing for a more differentiated assessment of HPV-related disease progression.

However, the main difference between our study and previous work lies in the methodology used to characterize the vaginal microbiome. Most contemporary studies, including Usyk et al. [25] rely on NGS, which amplifies and sequences hypervariable regions in the 16 S gene. This approach may yield uncertain species-level assignments, and bacterial frequency distributions vary depending on the region analyzed [27, 28]. More importantly, Usyk et al. [18]based their study on a molecular score for BV, although validated on standard measures such as Nugent score or Amsel criteria, although these two gold standards for BV diagnosis [8, 15] were not applied for their HPV-BV-data set.

In contrast we used a microscopic methodology to assess predominant vaginal colonization. This approach is consistent with the fact that bacterial vaginosis can be diagnosed using the Nugent procedure and the associated score [8]. It is therefore not at all surprising that microscopy generates results of comparable quality: a previous study demonstrated a strong correlation between the microbial profiled identified in Pap-smears and those obtained through NGS [23]. This supports our approach using the existing Pap-smear data. Our analysis included both conventional Pap smears and liquid-based cytology (SurePath) specimens; the distribution of these methods was not systematically recorded, and all samples were assessed microscopically following routine clinical procedures.

To date, microscopy-based longitudinal studies are scarce. Kero et al. identified BV as a covariate of HPV persistence but studied pregnant women, limiting generalizability [29]. Guo et al. reported a reduced HPV clearance in women with BV but did not evaluate cytological changes [13].

Therefore, to our knowledge this is the first longitudinal study to simultaneously evaluate cytological Pap smear findings, bacterial vaginosis, and HR-HPV infection dynamics.

Our findings indicate that a BV is a significant risk factor for the progression in Pap smears. This association between BV and dysplastic progression suggests that the vaginal microbiome plays a crucial role in establishing an environment that facilitates the persistence of HPV. These findings are consistent with previous research showing that the predominance of BV-associated bacteria, such as Gardnerella, is linked to persistence and progression in HPV infected patients and more generally that non-Lactobacillus dominated microbiomes carry a higher risk for progression [30] It has been suggested that this is caused by the potential immunosuppressive effects that Gardnerella may execute [31]. A possible mechanism may be a lowered immune response caused by environmental changes such as an increase in vaginal pH. As Clarke et al., 2012 demonstrated, there is a strong association between an increase in vaginal pH and the infection rate of HPV [32]. The protective properties of the vaginal mucosa may be reduced, and the local inflammation is increased by pro-inflammatory cytokines making it easier for the HPV to persist in the vaginal tract [33].

Conversely, a dominant Lactobacillus colonization has been identified as a protective factor exhibiting a significantly higher clearance rate. This suggests that a healthy vaginal flora offers protection against HPV and helps to clear the infection. A Lactobacillus dominated flora is not only a promising preventative strategy but may as well be a potential therapeutic avenue [34]. As shown, a transplantation of L. crispatus reduces the persistence rate of high-risk HPV infections [35]. Several different mechanisms are proposed to give Lactobacilli a beneficial role in vaginal health. This flora is thought to inhibit the growth of pathogenic vaginal bacteria by competing for adhesion sites on the epithelial layer and by producing lactic acid, thereby creating an unfavorable environment for pathogenic colonization [36].

While our findings provide relevant insights, limitations must be acknowledged: Although the microscopic detection of clue cells are a very strong indication for the presence of BV, this disease is usually diagnosed using the AMSEL criteria [15]; these were not available due to the retrospective study design. In addition, information on potential confounders other than age, e.g. sexual behavior or contraceptive use were not available. This may have influenced the observed associations.

In Germany the HPV screening strategy was revised in 2020 [37]. The current co-testing approach (HPV-PCR plus Pap smear every 3 years for women ≥ 35 years) provides higher sensitivity and specificity for detection of clinically relevant cervical lesions compared with cytology alone [38]. Given the high financial burden of the NGS method and the probable lack of its use for standard clinical screening in the near future, our study deliberately focused on Pap-smear based microbiological assessment, which is readily available within established cervical cancer screening programs. This may be a pragmatic tool for risk stratification in HPV-positive women and could help to further improve the effectiveness of screening programs as BV-positive patients may benefit from a closer follow-up period and earlier therapeutic intervention.

Further prospective studies in larger cohorts are required to validate our findings, ideally with shorter follow-up periods, especially for patients with BV. Future research should assess whether targeted treatment or modulation of the vaginal microbiome can influence HR-HPV persistence and progression, thereby informing more individualized screening and follow-up strategies.

## Conclusions

To sum up our findings, our study underlines the pivotal role of the vaginal microbiome and its potential effects on the natural history of HPV infections and associated cytological abnormalities. The significant association between BV and HPV progression in conventional pap smears suggests that interventions targeting the vaginal microbiome may be an important, yet underutilized, modifier of HPV-related disease outcomes. While further prospective studies are needed to clarify causality and the underlying biological pathways, our results indicate, that Pap smears routinely collected within screening programs provide a valuable opportunity to identify additional risk factors for HPV persistence and cytological progression. Respectively further studies could investigate whether BV diagnosed in pap smears could be treated effectively with the administration of probiotics containing lactobacillus or, if necessary, antibiotic intervention. Integrating microbiological assessment into existing screening strategies may ultimately contribute to more individualized follow-up and improved prevention of HPV-related disease progression.

## Supplementary Information

Below is the link to the electronic supplementary material.

Supplementary Material 1: Comparison of the Muenchner Nomenklatur 3 and the Bethesda System

Supplementary Material 2: Cohort A HPV-Categories in 2020

Supplementary Material 3: Cohort B HPV-Categories in 2021

Supplementary Material 4: Cohort A Data used for calculations

Supplementary Material 5: Cohort B Data used for calculations

## Data Availability

All data generated or analyzed during this study are included in this published article and its supplementary information files.

## Abbreviations

HPV: Human Papilloma Virus
CIN: Cervical intraepithelial neoplasia
NGS: Next generation sequencing
CST: Community state type
BV: Bacterial vaginosis
HR: High Risk
MN: Muenchner Nomenklatur III
OR: Odds Ratio
MWU: Mann-Whitney U
