# Supplementary material for: Bacterial vaginosis in microscopic examination of Pap smears in patients with high-risk HPV is associated with viral persistence and cytological progression in a longitudinal study
Source: Infect Agent Cancer. 2026 Jan 29;21:16. doi: 10.1186/s13027-026-00734-x (PMC12895659; doi:10.1186/s13027-026-00734-x)
Supplement: Supplementary file 1 — Supplementary Material 1: Comparison of the Muenchner Nomenklatur 3 and the Bethesda System [file 13027_2026_734_MOESM1_ESM.pdf]

Group	Definition	Correlation in Bethesda System
0	Insufficient material Repeat smear	Unsatisfactory for evaluation
I	Unremarkable and normal findings → next Smear in preventive screening interval	NILM
II-a	unremarkable findings with previous abnormal history → cytological follow-up due to abnormal history possible (cytological/histological/colposcopic/clinical findings)	NILM
II	Findings with limited protective value	
II-p	Squamous epithelial cells with mild nuclear changes such as CIN 1, often with cytolysis/parakeratosis → cytological control depending on clinical context (possibly after treatment for inflammation); additional methods and/or colposcopy in some cases	ASC-US
II-g	Glandular cells with atypia beyond the spectrum of reactive changes → cytological control depending on clinical context; additional methods and/or colposcopy may be necessary	AGC endocervical NOS
II-e	Endometrial cells in women >40 years, particularly in second half of cycle → Clinical control considering history and clinical findings	Endometrial cells
III	Unclear or ambiguous findings	
III-p	CIN 2 / CIN 3 / squamous cell carcinoma not excluded → Differential colposcopy, possible additional methods, short-term cytological follow-up after treatment of inflammation and/or hormonal regulation	ASC-H
III-g	Marked atypia of glandular epithelium, adenocarcinoma in situ/invasive adenocarcinoma not excluded → Differential colposcopy, possible additional methods	AGC endocervical favor neoplastic
III-e	Abnormal endometrial cells (especially postmenopausal) → Further clarification with clinical diagnosis, possibly histological assessment	AGC endometrial
III-x	Atypical glandular cells of undetermined origin → Further diagnostic work-up (e.g. fractional curettage); possibly differential colposcopy/additional methods	AGC favor neoplastic
IIID	Dysplasia findings with higher regression tendency	
IIID1	Cell image of mild dysplasia, analogous to CIN 1 → Cytological follow-up in six months; if persistent >1 year: further work-up/additional methods	LSIL
IIID2	Cell image of moderate dysplasia, analogous to CIN 2 → Cytological follow-up in 6 months; if persistent >6 months: further work-up/additional methods	HSIL
IV	High-grade precursors of cervical carcinoma	
IV-a-p	Cell image of severe dysplasia/CIN 3 / carcinoma in situ Differential colposcopy and treatment	HSIL
IV-a-g	Cell image of adenocarcinoma in situ	AIS
IV-b-p	Cell image of CIN 3, carcinoma in situ not excluded	HSIL with features suspicious for invasion
IV-b-g	Cell image of adenocarcinoma in situ, invasive cancer not excluded	AIS with features suspicious for invasion
V	Malignoma Further diagnostics with histology and therapy	
V-p	Squamous cell carcinoma	Squamous cell carcinoma
V-g	Endocervical adenocarcinoma	Endocervical adenocarcinoma
V-e	Endometrial adenocarcinoma	Endometrial adenocarcinoma
V-x	Malignoma of uncertain origin	Other malignant neoplasms
