# Supplementary figures and images for: Bacterial vaginosis in microscopic examination of Pap smears in patients with high-risk HPV is associated with viral persistence and cytological progression in a longitudinal study
Source: Infect Agent Cancer. 2026 Jan 29;21:16. doi: 10.1186/s13027-026-00734-x (PMC12895659; doi:10.1186/s13027-026-00734-x)

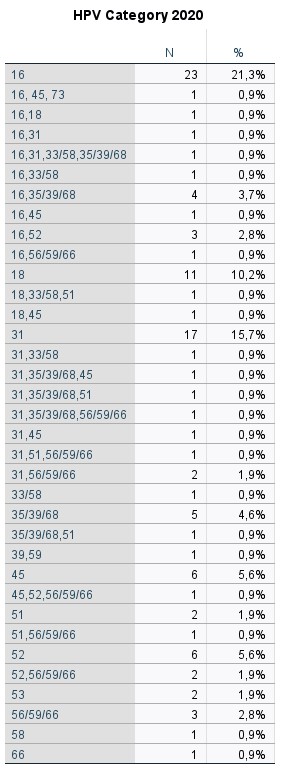

Supplement: Supplementary file 2 — Supplementary Material 2: Cohort A HPV-Categories in 2020 [file 13027_2026_734_MOESM2_ESM.jpg]

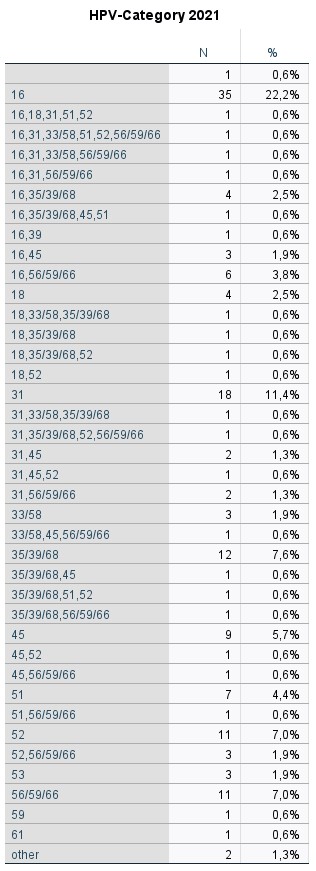

Supplement: Supplementary file 3 — Supplementary Material 3: Cohort B HPV-Categories in 2021 [file 13027_2026_734_MOESM3_ESM.jpg]
